# A chemical perspective on the chiral induced spin selectivity effect

**DOI:** 10.1093/nsr/nwae212

**Published:** 2024-06-21

**Authors:** Brian P Bloom, Zhongwei Chen, Haipeng Lu, David H Waldeck

**Affiliations:** Department of Chemistry, University of Pittsburgh, Pittsburgh 15260, USA; Department of Chemistry, The Hong Kong University of Science and Technology, Kowloon, Hong Kong 999077, China; Department of Chemistry, The Hong Kong University of Science and Technology, Kowloon, Hong Kong 999077, China; Department of Chemistry, University of Pittsburgh, Pittsburgh 15260, USA

**Keywords:** chiral, spin selectivity, hybrid organic-inorganic layered material, enantioseparation

## Abstract

This review discusses opportunities in chemistry that are enabled by the chiral induced spin selectivity (CISS) effect. First, the review begins with a brief overview of the seminal studies on CISS. Next, we discuss different chiral material systems whose properties can be tailored through chemical means, with a special emphasis on hybrid organic-inorganic layered materials that exhibit some of the largest spin filtering properties to date. Then, we discuss the promise of CISS for chemical reactions and enantioseparation before concluding.

## Teaser

Chiral induced spin selectivity (CISS) provides a new strategy for spin control in enantioselective chemical reactions and separations.

## INTRODUCTION

The control and manipulation of chemical processes have traditionally been performed through modulation of ‘classical’ parameters such as temperature, catalyst identity, bias voltage, etc. The chiral induced spin selectivity (CISS) effect is opening new possibilities for using the electron spin in chemistry [1]. Because magnetic interactions (Zeeman splitting) are small compared to Coulomb interactions, the electron spin is often viewed as only a minor contributor in chemistry, other than the accounting of electron populations in orbitals. The CISS effect shows, however, that the chiral symmetry (or structure) of molecules affects the transfer and displacement of electron spins in molecules; and because CISS involves spin-exchange interactions, it is large enough to affect reactions occurring at room temperature [2]. These facts suggest that CISS can have important effects on chemical reactions of chiral molecules and on the properties of materials which incorporate molecular chirality. In this review, we describe recent progress in charting how CISS can be used to affect chemical reactions and enantioselective separations.

**Figure 1.**
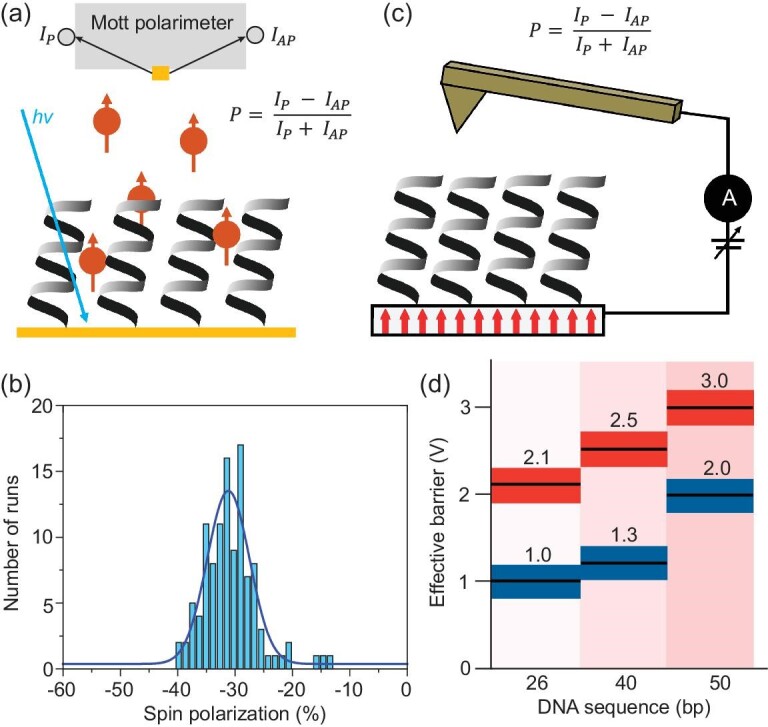
Panel (a) shows a schematic diagram for photoemission-based CISS measurements; excitation of the gold surface creates photoelectrons that transmit through a chiral monolayer and are then detected by a Mott polarimeter, which resolves them into parallel (*I*_P_) and antiparallel (*I*_AP_) polarizations. Panel (b) shows a plot of the photoelectron spin polarizations emanating from 50-base pair dsDNA coated gold substrates excited with linear polarized excitation. Panel (c) illustrates the experimental arrangement for mc-AFM measurements with a magnetized ferromagnetic substrate. Differences in the *I-V* characteristics with magnetization orientation are then monitored. Panel (d) shows the effective barrier determined from d*I*/d*V* data for Au nanoparticle–dsDNA–ferromagnetic substrate junctions that were measured using mc-AFM. The barriers were extracted for the electron spin aligned parallel (red) or antiparallel (blue) to the velocity. Panel (b) is replotted from data in Ref. [4], and Panel (d) is adapted from Ref. [6] with permission.

Early studies into the CISS effect used circularly polarized light to generate spin-polarized electron distributions and examined their transmission through chiral molecular films. The first study in 1999 by Ray *et al.* [3] measured how the yield of photoelectrons transmitted through Langmuir-Blodgett films of stearoyl lysine depended on the exciting light's polarization (clockwise or counterclockwise) and the molecules' chirality (*L*-lysine or *D*-lysine amino acids). For the Au films, the circularly polarized light creates a spin-polarized population of photoelectrons with their spin oriented anti-parallel (clockwise) or parallel (counterclockwise) to the electron velocity. For molecular films comprising *L*-stearoyl lysine layers, the detected photoelectron yield was higher for clockwise circularly polarized excitation than for counterclockwise excitation. Conversely, films of *D*-stearoyl lysine showed that clockwise circularly polarized excitation led to a lower photoelectron yield than counterclockwise circularly polarized excitation. The difference in photoelectron yield with excitation polarization was argued to arise from spin-dependent scattering through the chiral layers. In 2011, Gohler *et al.* [4] studied electron transmission through self-assembled monolayers (SAMs) of DNA and directly determined the spin of the photoelectrons through Mott polarimetry (see Fig. 1a for a schematic). Here, regardless of the excitation polarization, the intensity of electrons with their spins polarized parallel to their propagation direction was found to be larger than that for the spins polarized antiparallel to their propagation direction, unequivocally demonstrating that DNA acts as a ‘spin filter’; see Fig. 1b for the case of linear polarized excitation.

Although the early demonstrations of CISS focused on processes that occur above the vacuum level, in 2006 Wei *et al.* [5] reported CISS for electrochemical tunnel junctions. In this experiment, they used circularly polarized light to induce helicity in the excited electronic state of a porphyrin chromophore that was linked to the electrode by a chiral scaffold, and they measured how the photocurrent changed with the light's helicity and the enantiomeric form of the chiral linker. These early studies were followed by magnetic conductive atomic force microscopy (mc-AFM) measurements of molecular tunnel junctions. In mc-AFM, either the tip or the substrate is a magnetized ferromagnetic material that acts as a spin analyzer to quantify the spin-dependent current-potential (*I-V*) characteristics; Fig. 1c shows the case of a ferromagnetic substrate. The spin polarization is then determined by taking the difference in current measured when the electron transport is parallel (*I***_P_**) and antiparallel (*I*_AP_) and dividing by their sum (see inset of Fig. 1c). The effective barriers reported in Fig. 1d correspond to gold nanoparticle–double stranded DNA–nickel junctions with magnetized Ni substrates as a spin analyzer [6]. Interestingly, large differences in the current response were found when a North or South magnetic field was applied relative to the substrate, i.e. with electron transport parallel or antiparallel to its spin. The asymmetry in current response was used to extract an effective tunneling barrier and the barriers were found to increase with the increasing number of base pairs in the DNA duplex.

To date, several methods have been used to characterize the CISS effect, either directly or indirectly, and new types of measurements are continuing to be developed. The measurement approaches can be categorized into two general types: non-contact and contact methods. The non-contact methods include spin-resolved photoelectron spectroscopy (discussed above), time-resolved electron paramagnetic resonance (EPR) spectroscopy, and fluorescence spectroscopy methods [7]. Spin-resolved photoemission of electrons through a chiral molecular film can be directly analyzed by Mott polarimetry and was the original method to probe the CISS effect [4]. Recently, Wasielewski's group reported a direct observation of CISS in a donor–chiral bridge–acceptor molecule by time-resolved EPR spectroscopy [8]. Their results show that a solid (ferromagnetic) substrate is not required to observe the CISS effect from chiral molecules, much like that shown for donor-bridge-acceptor quantum dot assemblies that were studied using time-resolved fluorescence [9]. These contactless methods can directly probe the CISS effect without the influence of substrates and electrodes, but can require complicated spectroscopic setups and sophisticated data analysis. On the other hand, contact methods based on spin-resolved carrier transport are widely used to study the CISS effect. These approaches include mc-AFM [6], Kelvin probe force microscopy [10], magnetic force microscopy [11], spin valve devices [12,13], and spin-Hall effect devices [14]. Here, a ferromagnetic electrode or substrate is required to analyze the spin-dependent carrier transport.

Many newly discovered chiral materials, including chiral supramolecular assemblies and chiral organic-inorganic hybrid materials, have been studied by mc-AFM for CISS. However, a significant challenge in attaining a fundamental understanding of CISS lies in the difficulty of separating the role of the substrate from that of the chiral molecule. In addition, different methods are probing different domain sizes, which will impact the resulting spin polarization that is measured, as CISS is strongly affected by the structure, orientation, and defects of materials. Overall, it remains challenging to directly and reliably probe the CISS effect across different systems, and therefore, great care should be taken when comparing spin polarizations across different measurement methods.

The CISS field has experienced rapid growth in recent years. Whereas initial CISS measurements focused on small chiral organic molecules and biological supramolecules, such as DNA, amino acids, and oligopeptides, research now includes organized supramolecular structures, organic-inorganic hybrid materials, and transition metal dichalcogenides, possessing a wide range of conduction mechanisms. Moreover, studies are showing that the spin-mediated properties of chiral molecular materials actively contribute in biological, chemical, and physical processes [15]. Despite substantial experimental measurements on the CISS effect, a comprehensive theory which can account for the reported large spin polarizations is still lacking. Note that recent progress and development in our theoretical understanding has been reviewed elsewhere [16] and is beyond the scope of the current review. Instead, we focus our discussion on how chemistry can be used to engineer CISS, and how CISS can be leveraged to control and manipulate chemical processes.

## CHEMICAL CONTROL OF MATERIAL SYNTHESIS FOR CISS

In order to maximize the utility of CISS in chemical applications, it is imperative that we develop a diverse materials toolbox. For instance, the physical properties of metals and metal oxides are favorable for facilitating catalysis, whereas the large porosity of metal organic frameworks is well-suited for separations. Herein, we review selected classes of materials in which the properties are tailored, through chemical means, to create composites that display spin filtering. See Table 1 for a brief summary of different materials classes and their measured spin polarizations. These systems represent an ideal testbed for identifying key structure-CISS property relationships and for screening of material compositions with enhanced spin effects.

**Table 1.** Summary of spin polarizations reported from some different supramolecular chiral materials.

System	Compound	Spin polarization	Ref
Conjugated polymers	Zn-porphyrin dyes	50%	[17]
	Helicenes	45%–50%	[22]
	Self-assembled helicenes	80%	[23]
	Helically triphenylene-2,6,10-tricarboxamide polymers	40%	[24]
Organic-inorganic hybrid perovskites	(*R*-/*S*-MBA)_2_PbI_4_	92%	[28]
	(*R*-/*S*-MBA)_2_PbI_4_/CsPbBr_3_	80%	[43]
	(*R*-/*S*-MBA)PbBr_3_	90%	[37]
	(*R*-/*S*-MBA)_2_SnI_4_	94%	[36]
	(*R*-/*S*-MBA)_4_Bi_2_Br_10_	84%	[39]
	(*R*-/*S*-MBA)_2_CuBr_4_	92%	[40]
	(*R*-/*S*-MBA)_2_CuCl_4_	92%	[40]
	(*R*-/*S*-NEA)_2_CoCl_4_	90%	[38]
Metal-organic frameworks	Dy(III) *L*-tartrate	∼100%	[49]
	Cu(II)-(*D*/*L*-phenylalanine)	27% (*D*)/68% (*L*)	[50]
	Co(II)-(*D*/*L*-phenylalanine)	35%–45%	[51]
Transition metal dichalcogenides	(*R*-/*S*-MBA)-(H-TaS_2_)	63% (Magnetoresistance measurement)	[54]
	(*R*-/*S*-MBA)-(TiS_2_)	>90%	[56]
	(*R*-/*S*-MBA)-(MoS_2_)	75%	[55]

*R*-/*S*-MBA is *R*-/*S*-methylbenzylammonium; *R*-/*S*-NEA is *R*-/*S*-1-(1-naphthyl)ethylamine. Unless otherwise mentioned, all measurements are performed by mc-AFM.

### Chiral polymers and supramolecular assemblies

Systematic studies on chiral polymers and organized assemblies into chiral motifs have led to and/or support many of the physical notions known to affect the magnitude of the CISS effect. The CISS effect was first observed in these types of systems in 2017 by Mtangi *et al.* for helical aggregates of chiral Zn-porphyrin dyes, with mc-AFM spin polarizations as high as ∼50% at a −2.5 V bias [17]. Subsequent studies have shown that: (*i*) the magnitude of spin polarization correlates with the chiroptical properties determined through circular dichroism [18], a phenomenon akin to that first shown in chiral II–VI quantum dots [9], (*ii*) the spin polarization increases with increasing length of the material through which the electron traverses [19], consistent with that shown for DNA and oligopeptides [20], and (*iii*) the spin polarization in transport can persist for microns in length [21].

We note that point chirality is not necessary for observing the CISS effect. Initial studies demonstrated that the axial chirality in helicenes, molecules without stereocenters, is sufficient to produce spin filtering with polarizations on the order of 45%−50% [22]. In other works, researchers have self-assembled disubstituted helicenes into supramolecular polymers and observed marked enhancements in the spin polarizations (∼80%) [23]. A similar strategy was employed with achiral triphenylene-2,6,10-tricarboxamide monomers to create helically oriented supramolecular polymers that showed 40% spin polarizations [24]. Although assembled chiral molecules display various degrees of spin polarization, it remains challenging to establish a quantitative description of its origin. Models of CISS with simple spin-orbit coupling (SOC) predict orders of magnitude lower spin polarization than experimental observations, and it remains unclear how chiral secondary structures propagate spin polarization differently, and/or in conjunction with the isolated point chirality of the individual molecules. The precise synthetic control in these chiral materials can provide chemical knobs to tune structural and morphological properties systematically which will be crucial for establishing a quantitative structure-CISS relationship.

### Chiral hybrid metal halide semiconductors

**Figure 2.**
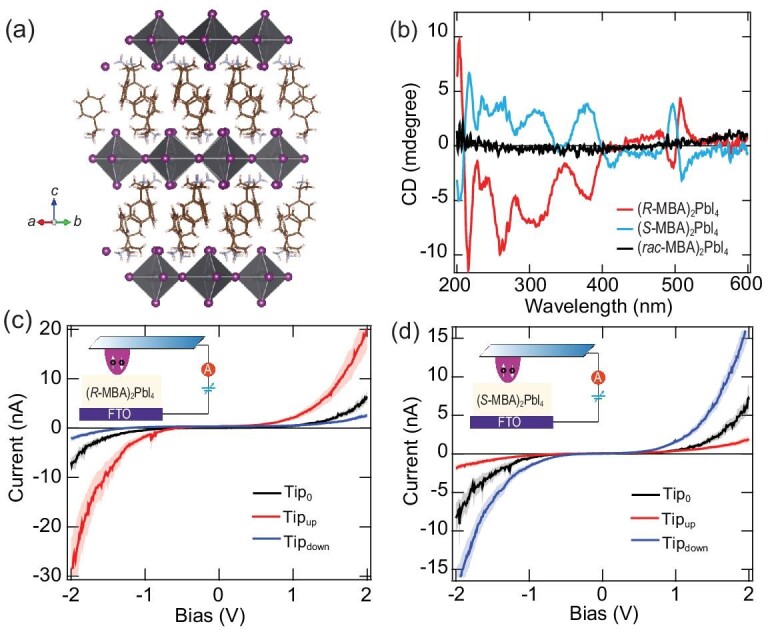
Panel (a) shows the crystalline structure of an (*R-*/*S*-MBA)_2_PbI_4_ perovskite, and panel (b) shows the corresponding circular dichroism spectra for the *R*- (red) and *S*- (blue) enantiomers, as well as their racemic mixture (black). Panels (c and d) show mc-AFM measurements on perovskites containing *R*- and *S*-MBA, respectively. The blue curves correspond to transport of carriers with their momentum parallel to the spin, and the red curves correspond to transport with the carriers antiparallel to their spin. The black curve is for measurements made with a non-magnetized tip. This figure is adapted from Ref. [28] with permission.

Hybrid metal halide semiconductors (MHSs) represent an attractive class of materials in optoelectronics, owing to their solution processability, versatile chemical diversity, tunable band gap, and high carrier mobility [25]. In addition, researchers have shown that incorporation of chiral organo-ammonium cations into MHSs imparts chirality [26,27]. Fig. 2a shows the crystal structure of (*R*-/*S*-MBA)_2_PbI_4_ [28–30], one of the most widely reported chiral MHS, which features a two-dimensional (2D) perovskite structure with chiral methyl benzyl ammonium (MBA^+^) as the templating cation. It was found that the asymmetric hydrogen bonding between the organic ammonium and lead halides gives rise to the Sohncke space group and chiroptical activity from inorganic subunits (Fig. 2b). Recent studies show that the optical activity of chiral perovskites can be modulated by the dimensionality, halide composition, and hydrogen bond interactions [31–35]. In 2019, Lu *et al.* reported the first observation of CISS in chiral (*R*-/*S*-MBA)_2_PbI_4_ thin films, where spin polarizations as high as 86% were observed by mc-AFM measurements (see Fig. 2c and d) [28]. Their subsequent studies on chiral (*R*-/*S*-MBA)_2_SnI_4_ achieved even higher spin polarizations (∼94%) [36]. Following these two works, other groups have reported relatively high spin polarization in various chiral MHSs [37–41]. The unique combination of solution processability, semiconducting properties, structural tunability, and high spin polarization make these chiral MHSs promising for constructing spin-based optoelectronics, for example spin-valves [28], spin-photovoltaics [42], and spin-light emitting diodes [43,44].

While the simple device fabrication of chiral MHSs significantly broadens the scope of CISS, it also brings forth challenges in our understanding of the CISS mechanism. The precise structure-CISS, structure-chiroptical, and chiroptical-CISS relationships remain unknown. Because most assembled MHS thin films reported so far appear to show large spin polarization and likely manifest collective effects, it has been difficult to isolate the contributions of different components in the performance directly. In addition, the spin polarization cannot be switched once the point chirality is fixed in chiral MHSs. Recent work shows that designer chiral organic components can be used to create chiral systems that can be controlled through external stimuli, i.e. light and/or temperature. For instance, Deng *et al.* synthesized 2D conformational chiral MHSs with (NH_3_(CH_2_)_2_SS(CH_2_)_2_NH_3_)^2+^(CystaH_2_^2+^) organic cations which undergo helical inversion, *P* to *M* upon application of an external electric field [45]. The chirality inversion in these systems is expected to give rise to switching of the CISS-mediated spin preference, similar to that shown with measurements on molecular motors [46].

### Chiral hybrid metal-organic frameworks

**Figure 3.**
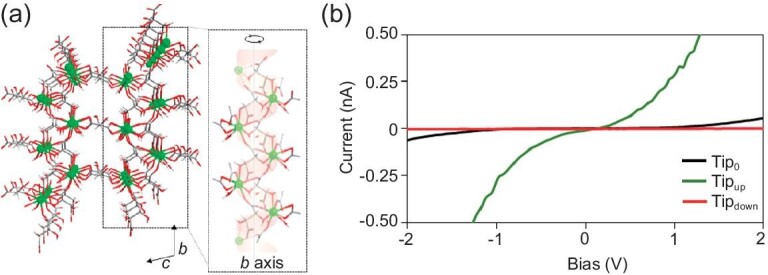
Panel (a) shows a representative crystallographic structure of the Dy(III) *L*-tartrate MOF. The right image shows the helicoidal secondary structure along the *b* axis. Panel (b) shows corresponding *I-V* curves from mc-AFM measurements on the Dy-L single crystal as a function of tip magnetization, electron transport parallel (green) and antiparallel (red) to its spin, and without an applied magnetic field (black). This figure is adapted from Ref. [49] with permission.

Chiral metal-organic frameworks have been developed and studied for asymmetric catalysis, enantioselective recognition and sensing, and enantioselective separations [47,48]; however, reports of the CISS effect in chiral MOFs, while compelling, are few. In 2020, Huizi-Rayo *et al.* synthesized 3D MOFs comprising Dy(III) and *L*-tartrate chiral ligands (Fig. 3a) which showed extraordinary spin polarizations, ∼100% (see Fig. 3b) [49]. In addition, the spin-polarized charge transport was found to occur over distances of ∼1 μm. In a similar vein, measurements on chiral Cu(II) phenylalanine crystals have been performed, and they exhibit CISS properties, albeit with lower spin polarizations [50,51]. Additionally, work reported by Goren *et al.* showed that chiral MOFs can be used to create spin transistor devices [52]. The spin transistor was found to exhibit nonlinear source-drain currents, with various states generated by the magnetization of the source. These pioneering works on chiral MOFs have set the stage for future studies which aim to combine the favorable features of MOFs in catalysis and separations with CISS.

### Chiral transition metal dichalcogenides

**Figure 4.**
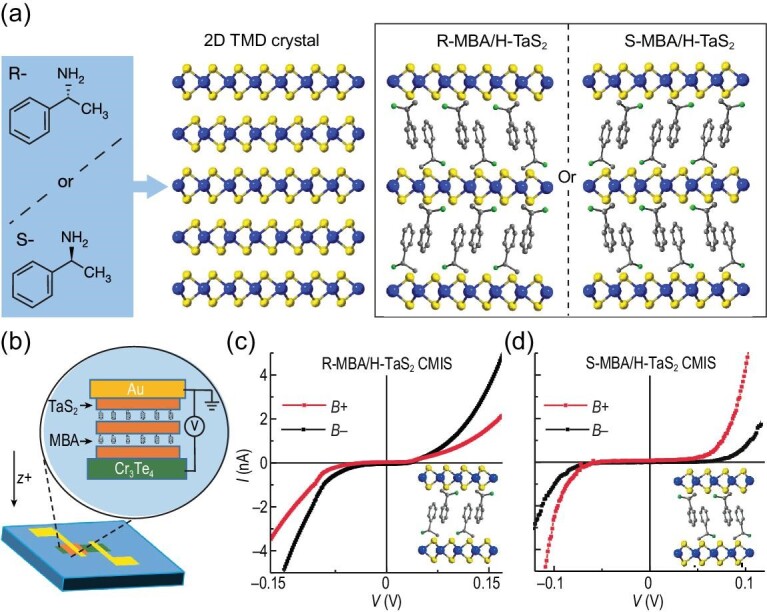
Panel (a) shows a schematic diagram for the intercalation of *R*- and *S*-methylbenzylamine molecules into a 2D H-TaS_2_ crystal; presenting alternating layers of monolayer TaS_2_ and chiral molecules. Panel (b) shows a diagram of the tunnel junction device used for measuring the CISS effect, and Panels (c and d) show corresponding *I*–*V* curves as a function of applied magnetic field with *R*- and *S*-MBA intercalators, respectively. This figure is adapted from Ref. [54] with permission.

Emerging synthetic strategies for intercalating chiral organic molecules into 2D atomic layers, such as transition metal dichalcogenides (TMDs), provide new opportunities for preparing artificial chiral solid-state materials [53]. TMDs possess non-bonding van der Waals interactions between covalent-bonded inorganic sheets, which enable incorporation of organic molecules into the gap without affecting the in-plane crystalline structure. Work by Qian *et al.* recently showed that chiral *R-*/*S*-methylbenzylamine organic molecules can be intercalated into H-phase tantalum disulfide (H-TaS_2_) through a straightforward wet chemical process (see Fig. 4a) [54]. The materials showed excellent spin-filtering properties, of ∼63%, in spin tunnel junction measurements. Fig. 4b shows the device geometry, and Fig. 4c and d show the corresponding *I-V* characteristics for TMDs with *R*- and *S*-chirality, respectively. Other chiral TMD structures have since been studied, e.g. TiS_2_ and MoS_2_, and show considerable spin polarizations (>75%) and spin persistence lengths (micrometers) [55,56].

### Summary and future outlook

Recent development of chiral materials for CISS has shifted from the early-stage chiral self-assembled monolayers (SAMs) to larger-scale assembled chiral organic or organic-inorganic hybrid systems, where the spin polarization is much higher. Although a predictive model for CISS is still lacking, features that give rise to large polarizations are becoming clear. For instance, in organic materials, highly ordered conjugated molecules and polymers exhibit much higher spin polarizations than equivalent randomly oriented counterparts. Likewise in hybrid materials, structural rigidity/crystallinity of the material is paramount for achieving large spin polarizations. As an example, chiral perovskite thin films show remarkably high polarization over the nano-sized domains measured by mc-AFM, but the polarizations measured in magnetoresistance device structures, which are micron in scale, is only about 1% [28]. This supposition is further corroborated by studies on single crystal materials that possess high spin polarization values over long transport distances [57]. Given these facts, hybrid organic-inorganic materials, such as chiral halide perovskites, are an important target for CISS based spintronic and optoelectronic applications, because they offer the benefits of superior structural rigidity and uniformity as compared to organic films and yet they allow for their properties to be tuned through the exquisite control over molecular properties that is available through organic chemistry. Nevertheless, the challenge of realizing uniform chiral molecule films and control over structural defects is daunting. Understanding these nuances, as well as others, will be important for realizing the true potential of CISS.

Despite the above challenges, applications of CISS in spintronics are expanding. The most direct application is the spintronic device [58], in which a ferromagnet layer is replaced by a chiral molecule layer whose spin polarization is induced by the CISS effect. Chiral-assembled molecules have been used in spin-valve devices [59] and spin memristors [60]. Diverse spin-optoelectronic devices [61], which control the spin degree of freedom of charge carriers, have been reported. One significant breakthrough is the demonstration of a room temperature spin light emitting diode (spin-LED) enabled by the CISS effect [43], where a chiral 2D perovskite (*R*-MBA)_2_PbI_4_ was used as the spin filter to produce spin-polarized carriers. The recombination of spin-polarized carriers in the achiral perovskite layer produces circularly polarized electroluminescence with 2.6% polarization at room temperature. Moreover, circularly polarized light photodetectors based on chiral perovskites have been reported to display large anisotropy factors owing to the CISS effect [62–65]. In addition, novel superconducting spintronics [66] and quantum spintronics [67,68] based on CISS effects have also been reported.

From the perspective of materials development, there seems to be tension in the observation and understanding of CISS. On one hand, assembled chiral systems show a more pronounced CISS effect than that of chiral molecules. On the other hand, the collective nature of spin transport measurements of macroscopic assembled systems makes it more challenging to study the microscopic mechanism underpinning CISS. Thus, future studies are likely to continue developing assembled chiral systems for spin-based applications, whereas fundamental studies into the mechanism of CISS are more likely to arise from studies on simpler chiral systems, molecules.

## CHEMICAL IMPLICATIONS OF CISS

**Figure 5.**
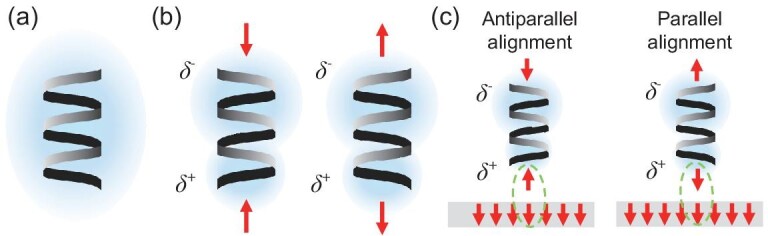
Panel (a) shows a representative closed shell molecule in which the molecule's chirality is indicated by the handedness of the helix with the blue region representing the electron cloud. Upon charge polarization, the electron redistribution in the chiral molecule causes an enantiospecific spin polarization, which is shown by the red arrows in panel (b), for a left-handed helix (left) and a right-handed helix (right). When a chiral molecule interacts with a spin-polarized surface (Panel (c)), the spin exchange interaction is enantiospecific; the interacting spins (green dotted line) are aligned parallel or antiparallel with the surface electrons.

The strong spin filtering that arises in chiral organic-inorganic materials underscores the important link between chirality and the electron spin, which CISS embodies. While the creation of chiral structures that control electron spin properties is an important research avenue, the use of electron spin to affect the synthesis of chiral molecules (and chiral materials) is equally important. How does the CISS effect manifest in chemical processes? To understand this, we must first understand the manifestations of CISS beyond that of spin selective transport. Consider a chiral molecule, represented by a helix in Fig. 5a. Even if the molecule is closed shell, upon charge polarization the redistribution of the electron cloud (blue oval) inside the molecule generates a transient spin polarization (Fig. 5b) [69]. Note, the charge redistribution of the molecule can arise from intermolecular collisions or by approaching a surface. The charge polarization induced spin polarization gives rise to enantioselective interactions between a chiral molecule and a spin-polarized surface or molecule. Fig. 5c illustrates this mechanistic process for chiral molecules at a magnetized substrate; the opposite spin polarization expressed by the two enantiomers leads to differences in the molecule-substrate spin exchange interactions. Such behavior forms the basis of CISS-mediated enantioseparations and electrochemical enrichment.

It should be noted that a comprehensive theory for CISS effects remains to be developed. While early theoretical models used a single electron picture with a parameterized spin-orbit coupling (SOC) to account for spin filtering, more recent models employ many body interactions or spinterface effects to account for the large SOC magnitudes that are needed to rationalize the experiments. In other efforts, Wolf *et al.* proposed that chiral molecules act as a spin polarizer rather than a spin filter, in which both the molecule chirality and current direction contribute to the spin polarization direction [70]; and Yang *et al.* proposed a spin-dependent electron transmission model for the CISS effect [71].

### Enantioseparations

**Figure 6.**
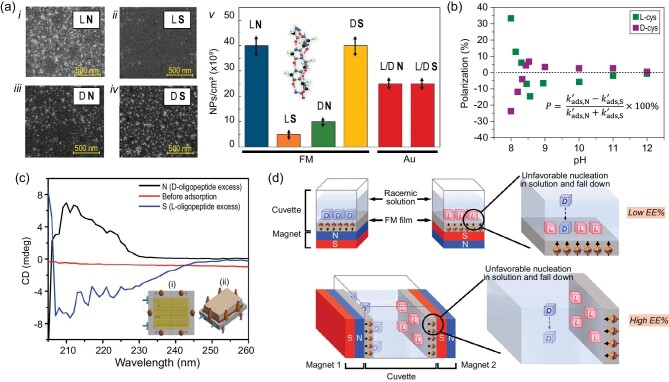
Panel (a) shows adsorption of polyalanine oligomers (molecular structure is shown in the inset of *v*) onto magnetized ferromagnetic substrates. SiO_2_ nanoparticles were attached to the adsorbed oligomers to quantify the coverage of *L*-polyalanine oligomers (*i* and *ii*) and *D*-polyalanine oligomers (*iii* and *iv*) with the magnetic dipole pointing North (N, *i* and *iii*) or South (S, *ii* and *iv*). A summary of the coverage on ferromagnetic substrates and control experiments on nonmagnetic substrates is shown in *v*. Panel (b) shows magneto-electrochemical quartz crystal microbalance measurements for determining the polarization in adsorption rate constant with magnetic field of *L*-cys (green) and *D*-cys (purple) molecules. Panel (c) shows circular dichroism spectra of a racemic mixture of alpha helical oligopeptides at the inlet (red) and outlet (blue, black) of a flow cell constructed on a ferromagnetic substrate. The sign of the CD, positive or negative, and hence enantiomeric excess depends on the magnetization, N or S, respectively. The inset of the figure shows a top down (*i*) and side view (*ii*) of the flow cell. Panel (d) shows a vertical (top) and horizontal (bottom) design strategy for the resolution of racemic solutions using CISS. Panels (a and c) are adapted from Ref. [72], Panel (b) is replotted from Ref. [75], and Panel (d) is adapted from Ref. [80] with permission.

The resolution of a pair of enantiomers using CISS was first demonstrated by Naaman and coworkers for cysteine, polyalanine, alpha helical oligopeptides, and dsDNA at a gold-coated ferromagnetic substrate [72]. Fig. 6a shows measurements in which magnetized ferromagnetic substrates were dipped into enantiopure solutions of polyalanine (either *L* or *D*) to which SiO_2_ nanoparticles were attached, to act as a marker for determination of the density through scanning electron microscopy (SEM). The corresponding SEM images of *L*- (*i* and *ii*) and *D*- (*iii* and *iv*) polyalanine were collected on ferromagnets with their magnetization pointing North (N, *i* and *iii*) and South (S, *ii* and *iv*). A summary of the resulting densities (*v*) along with control experiments on gold substrates is also shown. These data show that *L*-polyalanine preferentially adsorbs onto H+ magnetized substrates, whereas the *D*-polyalanine preferentially adsorbs on S magnetized substrates. Note that no dependence on magnetic field was observed for non-magnetic (gold) substrates, illustrating the importance of spin exchange in the mechanism.

The magnitude and sign of the spin polarization generated in a molecule upon charge polarization are sensitive to the solution conditions, adsorption geometry, and dipolar field [73–76], so that the spin exchange interaction, and hence the enantiopreference, in the separation can be manipulated. For example, Lu *et al.* used magneto-electrochemical quartz crystal microbalance methods to study the enantioselectivity of cysteine, quantified by the asymmetry (polarization, *P*) in adsorption rate constant with magnetization orientation of a ferromagnetic substrate, as a function of solution pH (see Fig. 6b) [75]. For *L*-cysteine, a strong positive polarization is observed at pH 8; however, at pH 8.5 the polarization changes sign, i.e. the adsorption process prefers the opposite magnetization, before finally plateauing at higher pHs. The crossover point in the polarization coincides with the pKa of cysteine and implies that changes in the binding orientation of the molecule result in an opposite spin exchange interaction. Enantiospecific interactions between ferromagnetic surfaces and chiral molecules have also been observed with other chiral molecules; *n*-acetyl cysteine, 1-amino-2-propanol, histidine, proline, and 1-phenylethanol [73,77,78].

To implement an enantioselective separation system based on these principles, a flow cell apparatus was developed using a magnetized ferromagnetic substrate. The inset of Fig. 6c shows a top view (*i*) and side view (*ii*) of the apparatus [72]. Circular dichroism measurements of a racemic solution of alpha helical peptides before (red curve) and after transiting through the flow cell (black, blue) illustrate that an enantiomeric excess is generated with a sign that depends on the magnetization of the ferromagnetic substrate. In addition, researchers have also begun to explore how CISS-mediated spin exchange interactions could be employed in column chromatography using Janus magnetic microparticles [78].

The enantiospecific interaction between chiral molecules and magnetized ferromagnetic surfaces can also act to seed the enantiomeric resolution of conglomerates through crystallization. Tassinari *et al.* first demonstrated this strategy for the crystallization of asparagine and glutamic acid, achieving an enantiomeric excess of ∼60% [79]. Upon improvement of the separation design from a vertical geometry, in which nucleation of the unfavorable enantiomer can ‘drop’ onto the surface, to a horizontal geometry, in which the unfavorable enantiomer deposits on the bottom of the container, much higher enantiopurity was achieved, ∼95% [80]. See Fig. 6d for a schematic representation of the different experimental designs for separation. In addition to asparagine and glutamic acid, chiral resolution through crystallization of threonine, imeglimin, and ribo-aminooxazoline have been performed [79–81].

### Enantioselective chemistry

**Figure 7.**
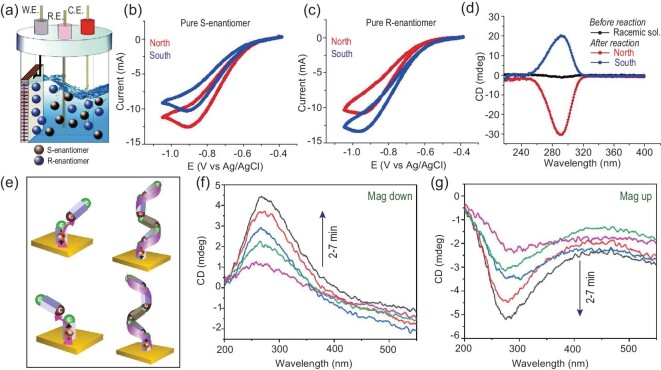
Panel (a) shows a schematic diagram illustrating the experimental approach for performing enantioselective electrochemistry at a ferromagnetic electrode. Panels (b and c) show the electroreduction of an enantiopure solution of *S*- and *R*-camphorsulfonic acid, respectively, with a ferromagnetic working electrode magnetized North (red) and South (blue). Panel (d) shows the circular dichroism spectra of a racemic solution of camphorsulfonic acid before (black) and after electrolysis with a ferromagnetic electrode magnetized North (red) and South (blue). Panel (e) shows the mechanistic process of forming chiral polymers with achiral monomers and spin-polarized electrons which determine the orientation-dependent coupling between a monomeric unit in solution to that of an initiation site on the electrode. The top part of the figure shows the case for spin down polarized electrons and the bottom part of the figure shows the case for spin up polarized electrons. Panels (f and g) show circular dichroism spectra for the electropolymerization of 2-vinylpyridine as a function of polymerization time on down and up magnetized electrodes, respectively. The figure is adapted from Refs [77] and [19] with permission.

The same guiding principles for enantioseparations can also be applied to facilitate electrochemically-driven, enantioselective redox chemistry. Fig. 7a shows a general scheme for an experiment in which the spin-dependent exchange interactions between chiral molecules and a magnetized ferromagnetic electrode manifest as changes in the current response [77]. Fig. 7b and c show cyclic voltammograms for the electrochemical reduction of enantiopure camphorsulfonic acid solutions at a ferromagnetic electrode with North (red) or South (blue) externally applied magnetic fields. For the *S*-enantiomer, a higher current is observed for a North applied magnetic field, whereas the opposite is true for the *R*-enantiomer; a higher current is observed for a South applied magnetic field. In this study, the difference in reactivity for the two enantiomers was used to electrochemically enrich a racemic solution. Fig. 7d shows circular dichroism spectra of a racemic solution before (black) and after electrolysis for six hours with application of a North (red) or South (blue) applied magnetic field. The intensity corresponds to an enrichment of ∼10%. As with the separations strategy described above, the design for the reaction mixture, represented by Fig. 7a, leads to a moderate enantiomeric excess.

Interestingly, electroorganic synthesis of prochiral reactants, namely in the electrooxidation of methylphenylsulfide and in a Diels-Alder cycloaddition of 3-dimethylbutadiene with acetaldehyde, at magnetized ferromagnetic electrodes have also been shown to give rise to products with an enantiomeric excess [82]. Although an exact mechanism for the process has not yet been determined, the dependence of the preferred enantiomer on the magnetization orientation of the substrate suggests that CISS is responsible for the enantiopreference.

Creation of a new stereocenter during a CISS-controlled chemical reaction has also been reported for the electropolymerization of 2-vinyl pyridine [19]. Here, the spin-polarized electrons, emanating from the magnetized ferromagnetic surface affect the orientation-dependent coupling between a monomeric unit in solution to that of an initiation site on the electrode (see Fig. 7e). The geometry of the resulting dimer/short chain oligomer acts to propagate the spin polarization from the electrode and ‘locks in’ the local chirality during polymerization. Fig. 7f and g corroborate this hypothesis by showing that the intensity of the chiroptical features of the polymer increase with polymerization time. In related work with chiral monomeric units, researchers have shown that the magnetization of ferromagnetic substrates relative to the enantiomeric form of the monomer can govern the rate of electropolymerization, a process attributed to spin exchange interactions [83]. Other reports have shown that chiral secondary structures without stereocenters can result in electropolymerization at ferromagnetic surfaces, e.g. with pyrenecarboxylic acid, carbazole, and 3,4-ethylenedioxythiophene monomers [77,84]. A similar phenomenon was also reported by Aizawa *et al.* for the organization of achiral cobalt phthalocyanines into helical supramolecule structures on ferromagnetic substrates upon crystallization [85].

### Spin-selectivity improves electrochemical selectivity

**Figure 8.**
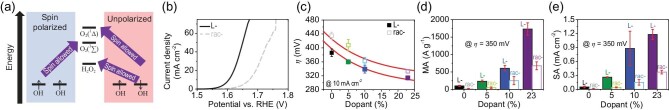
Panel (a) shows an energy diagram illustrating the possible reaction pathways for the combination of hydroxyl radicals on a catalysts’ surface. Formation of triplet oxygen O_2_(^3^∑) is promoted on a spin-polarized surface, whereas the singlet byproduct H_2_O_2_ is inhibited. Panel (b) shows linear sweep voltammograms of cobalt oxide catalysts synthesized with *L*- (black) and rac-cysteine (gray). Panel (c) plots the overpotential of a series of *L*- (filled symbol) and rac- (open symbol) iron doped cobalt oxide catalysts. Panels (d and e) plot the corresponding mass activity and specific activity, respectively. The figure is adapted from Ref. [91] with permission.

Work on the oxygen evolution reaction (OER) and the oxygen reduction reaction (ORR) shows that spin currents from electrodes can affect the reaction pathways when changes in electronic state multiplicity occur. For chemical reactions involving oxygen, the importance of the CISS effect is attributed to O_2_ being a triplet in its ground state [86]. The formation of O_2_ in the OER involves the coupling of oxy/hydroxy radical intermediates, and it is favored when the intermediates are spin aligned (see Fig 8a) [87–89]. Similarly, the reduction of O_2_ is enhanced with spin alignment between the oxygen and the catalyst [90]. Because CISS acts to spin-polarized redox reactions and provides spin-polarized catalytic surfaces, a favorable environment for spin alignment emerges. Such behavior leads to a reduction in the reaction overpotential, so much so that a change in the rate-determining step has been observed [91], as well as an improvement in the Faradaic efficiency by inhibiting singlet mediated byproduct formation [9]. Note that a similar enhancement in catalytic activity can occur for OER and ORR on magnetized ferromagnetic substrates [92–95]; however, larger improvements are observed when spin alignment is facilitated through CISS, likely owing to the higher spin polarizations [96].

Fig. 8b illustrates a representative example of OER enhancements attributed to CISS for cobalt oxide catalysts [91]. Here, linear sweep voltammograms for chiral catalysts (black) exhibit a much lower overpotential, *η*, than analogously prepared racemic catalysts (gray). Upon doping the catalysts with iron, a decrease in *η* (at a current density of 10 mA cm^−2^) is observed for both chiral (filled symbols) and racemic catalysts (open symbols). The chiral catalysts, however, consistently deliver superior performance (see Fig 8c). The mass activity (MA), in which the current is normalized to the mass of the catalyst, and specific activity (SA), in which the current is normalized to the electrochemical surface area, is plotted at an overpotential of 350 mV in Fig. 8d and e, respectively. The much larger MA and SA observed for chiral catalysts compared to racemic catalysts illustrate that CISS improves OER performance metrics.

CISS-enhanced oxygen electrocatalysis has now been observed using chiral organic supramolecular structures [17,97,98], metal oxides [89,91,99–103], and metal sulfides [55,104], among others [105–108], and CISS may contribute to the high activity observed in photosystem II [109]. The benefit of spin polarization-promoted catalysis has been shown for catalysts or electrodes coated with chiral molecules [90,101,110,111], chiral inorganic solids which do not contain organic molecules [89,99], as well as chiral materials that act as spin transport layers between the catalyst and the electrode [96,112]. Moreover, the favorable effects of CISS have been shown to persist for photoelectrochemical processes [112,113]. The compelling results of spin-enhanced catalytic activity for O_2_ reactions suggests that other types of reactions that involve radical intermediates, such as the carbon dioxide reduction reaction and nitrogen reduction reaction [114], may also benefit from the incorporation of chiral spin filtering properties into the electrocatalysts. Indeed, recent work on the carbon dioxide reduction reaction attributes the spin polarizations emanating from chiral catalysts, i.e. the CISS effect, for directing the reaction pathway from key intermediates toward more value-added products [115].

### Summary and future outlook

Proof-of-principle experiments have now unambiguously demonstrated that CISS can impact separations and chemical reactions. Although different models have been proposed to explain the CISS effect [15,116], gaps in our understanding are significant. Quantitative discrepancies between theoretical predictions and experimental data remain. The relationship between the chiroptical activity and CISS is not yet revealed, but promises important insights if it can be. Temperature-dependent CISS studies may also provide an important pathway to understanding and explaining the CISS effect, or at least the role of vibrations and phonons in CISS. The future development of theoretical models of CISS should be directed toward answering these important questions.

To elucidate and optimize the working principles underpinning CISS, more refined measurements and techniques are required. Electrochemical quartz crystal microbalance [73,75,76] and spin exchange microscopy measurements [117] are providing useful information on the exchange interactions between spin-polarized species which drive CISS-mediated separations. Numerous groups have now demonstrated the benefit of exploiting the spin filtering properties of chiral electrocatalysts for redox chemistry involving oxygen, and work toward extending this strategy to other redox reactions is beginning to appear. While CISS-based spin filtering has been shown to affect enantioselectivity, the enantiomeric excess (ee) values that have been reported are modest and much work remains to be done.

**Figure 9.**
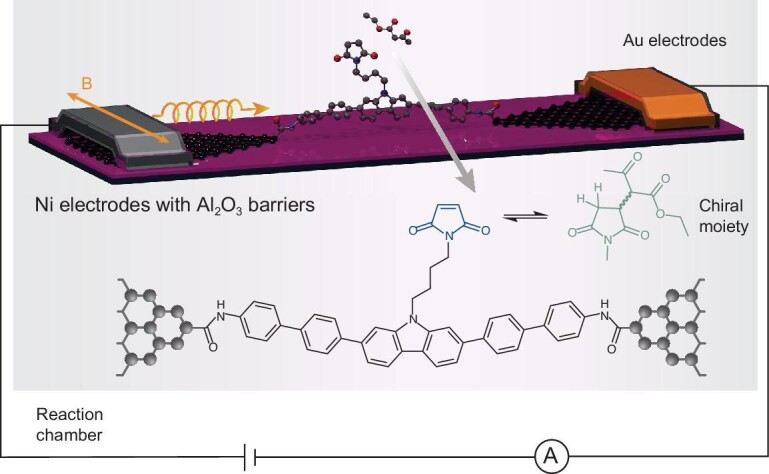
Schematic of a single-molecule spin valve device used to monitor the emergent chirality in a Michael addition reaction involving 1,3-dicarbonyl compounds with maleimide. The figure is adapted from Ref. [118] with permission.

Most efforts have used the CISS effect to direct chemical reactions; however, recent work by Yang *et al.* takes a different approach and uses chirality-based spin filtering to monitor an enantioselective reaction (Fig. 9) [118]. They reported the direct monitoring of chirality variations during a Michael addition of 1,3-dicarbonyl compounds to maleimide through a single-molecule junction device with the structure Ni/Al_2_O_3_/graphene/single molecule/graphene/Cr/Au, in which the ferromagnetic Ni layer provides the polarized spin injection. Continuous current measurements in the single-molecule junction revealed *in situ* chirality variations during the reaction, which follow the behavior expected for the CISS effect and further substantiate that CISS occurs at the single-molecule level. These interesting results, however, are being questioned in the literature, and it seems that further work is required to substantiate the conclusions [119]. Experimental platforms such as these promise fruitful discoveries on the importance of electron spin in controlling and manipulating chemical processes.

## CONCLUSION

The essence of chemical processes lies in the breaking and formation of chemical bonds, which are the result of electron interactions. Many important chemical reactions and catalytic processes involve reaction intermediates, such as open-shell complexes or radicals, where electron spin states can play an essential role in determining the reaction rate and pathway. The CISS effect represents a novel strategy for controlling the spin polarization of surfaces and molecular species and therefore can be used to facilitate, or inhibit, chemical processes. Implementation of the CISS effect, beyond that of simple demonstrations of its capabilities, however, requires the development of chiral materials with large polarizations. The identification and understanding of principal structural and electronic features which lead to strong CISS properties are thus of great importance for advancing the field.
